# The Origin of Cow-Pox and the Nature of Vaccine Virus

**Published:** 1864-09

**Authors:** 


THE ORIGIN OF

### COW-POX AND THE NATURE OF VACCINE VIRUS.

Investigation on this subject in the Paris Academy of Medicine, has led to the following conclusions:—

1st. That vaccine virus (as a thing separate and apart) has no existence.

2d. That the pretended vaccine virus, which we consider antagonistic to, and neutralizing the variolus virus, is the variolus virus itself.

3d. That the equine and bovine species are subject to an eruptive malady which is identical, as regards its nature, with variola of the human species.

4th. It is demonstrated that the same is the fact as regards several other species of animals, pigs, sheep, dogs, goats, etc.

5th. The local and general phenomena with animals are the same as those observed in man. The only difference as regards the pustules are those which depend on the structure of the skin and the number of the hairs.

6th. As in the human species, so in the equine and bovine, variola may appear sporadically.

7th. From the horse we may inoculate the cow, and reciprocally.

8th. From the cow we may inoculate, without difficulty, individuals of the human species, provided they have not had spontaneous or inoculated variola.

9th. The cow, the horse, and several other species may be inoculated with variolous matter from the human species.

10th. When a variolous epidemic occurs among men, it often extends itself, by contagion, to other animals.

11th. An epidemic of variola may commence among animals, and extend to man.

12th. Inoculated variola produces a much less degree of general reaction than does variola developed by contagion. This is true in both man and lower animals.

13th. The pustules which result from inoculated variola, are often limited to the points inoculated.

14th. When a secondary eruption is produced, it is almost always insignificant, and composed of a small number of pustules.

15th. In a general manner we may say that the variola of animals is more discrete, and less severe, than that of the human species.

16th. That the dangers of inoculation of variola in man have been much exaggerated. The unprejudiced study of what has been written on this subject will convince of this.

17th. It is probable that animals, as man, are subject to aphthous eruptions.

18th. But the *maladie aphtheuse*, as it is described by writers on veterinary medicine, is nothing less than variola.—*Medico-Chirurgical Review.*

---

*Dissolving Power of the Pancreatic Juice.*—M. Corvisart has shown that in animals the pancreatic juice has the power of dissolving albuminous foods without the assistance of the gastric juice or the bile; and now he has demonstrated the same thing in man. A hospital patient, in perfect health, having suddenly died from chloroform administered for the reduction of the femur, M. Corvisart removed the pancreas; and with the prepared juice and ferment of it, operated on albuminous matters. He found that a large quantity of albumen and fibrin was rapidly digested with its assistance.—*Brit. Med. Jour.*, May 28, 1864.

---

*Aggregate Labor of Mankind.*—Along with the compassion that is excited by listening to a tale of want, there is apt to arise, at the same time, a feeling of astonishment that such a thing should *be* in a land like this. Perhaps, however, the true wonder is that want is not universal. One-half of the race die before they have contributed an iota to the world's sustenance or their own. One-half of those who survive the period of childhood are women, who do not, as a general thing, contribute directly to the production of wealth. Of the men, many are sick, many are old, many are idle, many are wasteful, many are parasites. Those who do work and live to the age of three score years and ten, spend one-third of their lives in bed, one-twentieth at the table, one-sixth in recreation. Much of their time is wasted in mistakes; much of what they succeed in producing is swept away by fire and flood. During half of the year Nature sleeps. One harvest in five proves a failure. Only a fraction of the earth's surface is capable of cultivation. A large part of the general labor is absorbed in the production of luxuries, in repairing the damages of war, in preparing for future conflicts, in the transportation of produce, and in journeys. Probably not more than *one-tenth* of the whole amount of human force is expended in earning the world's daily bread. The standing marvel, therefore, of society is, not that any should suffer want, but that there should be any who do not.—*Home Journal.*

---

*Atropia : Its Action and Uses.*—Atropia, when applied locally in solution (without spirit), is not a local paralyzer, as commonly supposed, but it appears rather to quicken the contraction of the parts to which it is applied. Applied directly to the smaller arteries, atropia constricts them (Jones), and this effect may continue for many hours. It hence, probably, causes relaxation of the tissue of the iris, by causing constriction of the arteries entering it. In an atropised eye, the vision is clearer with distant than with near objects; this shows that the lens has receded and approached the retina, and is caused by a relaxation of the erectile structure of the ciliary processes from the constricting action of the atropia. This theory of the turgescence or emptiness of the vessels of the iris, causing, respectively, the contraction or dilatation of the pupil, explains why we have wide pupil so frequently in atonic and exhausting diseases, in aneurism of the chest and neck, impeding the passage of blood into and through the carotid. Worms and diseases of the belly in children dilate the pupil and impair the sight, probably by reflex action through the sympathetic nerve, exciting the radial fibres of the iris, and contracting the cerebral arteries.—*Dr. A. Fleming.*

---

*Grafting Animals.*—Dr. Paul Bert has published a work on the curious subjects of animal grafts. He succeeded in making Siamese twins of a couple of rats, and many other monstrosities. He exclaims, "it is a surprising spectacle to see a paw cut from one rat, live, grow, finish its ossification, and regenerate its nerves under the skin of another, and when we want a plume of feathers under the skin of a dog, what a miracle to see the interrupted vital phenomena resume their course, and the fragment of a bird receive nourishment from the blood of a mammal.—*Intellectual Observer.*

*Deaths From Chloroform.*—An inquest was held on Friday, June 24th, at King's College Hospital, on the body of Mrs. E. Ruth, aged 29, who died from the effects of the vapor of chloroform during an operation for removing a tumor in the urethra. A question arose as to the quantity administered to her on a former occasion, and the absence of books of reference in such cases in the hospital. The following verdict was returned:—"That the deceased died from the effects of the vapor of chloroform," and the jury expressed their opinion that proper case-books for reference should be kept in the hospital. Another fatal case occurred at Middlesex Hospital, on Tuesday, July 5th. This was an operation for the removal of a large tumor from the face, during which the patient died on the operating table. A third case occurred at St. Mary's Hos pital, where an inquest was held on Monday, July 18th, on the body of James Birch, aged 56, who died while under the influence of chloroform, administered previous to an operation for the removal of the bone of the great toe. A *post mortem* showed extensive disease of the heart.—*London Phar. Jour.*

